# Editorial for the Special Issue “Skin and Cutaneous Adnexal Tumors: Diagnosis and Management”

**DOI:** 10.3390/diagnostics14050554

**Published:** 2024-03-05

**Authors:** Dimitra Koumaki

**Affiliations:** Dermatology Department, University Hospital of Heraklion, 71110 Heraklion, Greece; dkoumaki@yahoo.gr; Tel.: +30-6974783589

We are delighted to present the Special Issue on “Skin and Cutaneous Adnexal Tumors: Diagnosis and Management” in *Diagnostics*. This collection of articles encompasses a comprehensive exploration of various aspects surrounding the diagnosis, pathogenesis, and management of skin and cutaneous adnexal tumors, offering valuable insights for clinicians, researchers, and pathologists [1,2].

The first article by Ferronika et al. (contribution 1) focuses on basal cell carcinoma (BCC), the most prevalent skin malignancy worldwide. Through meticulous profiling of tumor-infiltrating lymphocytes (TILs) and regulatory T cells (Treg cells) in different tumor zones, the study sheds light on the immunological dynamics within BCC. Notably, a high proportion of Treg cells and a relatively low count of TILs in the tumor microenvironment underscore the complexity of immune regulation in BCC progression.

Kyrmanidou et al. (contribution 2) delve into a study of eccrine poroma (EP), an enigmatic benign adnexal neoplasm with clinical and dermoscopic variability, often posing diagnostic challenges. By synthesizing current data on its pathogenetic mechanisms and diagnostic tools, the authors navigate through the intricate landscape of EP diagnosis and management, emphasizing the importance of novel diagnostic modalities in overcoming diagnostic dilemmas.

Apalla et al. (contribution 3) broaden the scope to encompass cutaneous sarcomas (CS), a heterogeneous group of rare mesenchymal neoplasms. Through a meticulous review of clinical and dermoscopic characteristics, the article delineates patterns that may aid in the early identification of CS, paving the way for timely intervention and improved outcomes. The CS discussed include dermatofibrosarcoma protuberans (DFSP), atypical fibroxanthoma (AFX), cutaneous undifferentiated pleomorphic sarcoma (CUPS), Kaposi’s sarcoma (KS), cutaneous leiomyosarcoma (CLMS), and cutaneous liposarcoma. These tumors present challenges in diagnosis due to their rarity and variable clinical presentation. Dermoscopy, a non-invasive diagnostic technique, is explored as a tool to aid in the early identification of CS. DFSP, the most common CS, presents as a firm plaque with nodules and exhibits specific dermoscopic features such as a pink background, depigmented areas, and linear vessels. AFX primarily affects the elderly and displays unspecific dermoscopic patterns resembling other malignant tumors. CUPS, a rare soft tissue sarcoma, presents as a subcutaneous nodule and shares nonspecific dermoscopic features with poorly differentiated squamous cell carcinoma. KS, associated with the HHV8 virus, presents heterogeneously and exhibits varied dermoscopic patterns corresponding to different clinical morphologies. CLMS, an aggressive tumor, presents as a dermal nodule and shows specific dermoscopic features including linear or circular structures with white to yellow coloration. Cutaneous liposarcoma, extremely rare, presents as a subcutaneous nodule and lacks specific dermoscopic patterns. The article emphasizes the importance of dermoscopy in conjunction with the clinical context for suspicion of CS, as it may aid in early diagnosis and prompt management. However, due to the rarity of some CS types, further research is needed to establish specific dermoscopic patterns for accurate diagnosis.

Papadimitriou et al. (contribution 4) provide a comprehensive overview of sebaceous neoplasms, ranging from benign lesions to malignant counterparts, with a particular focus on sebaceous carcinoma and its association with Muir–Torre Syndrome (MTS). By elucidating the clinical, dermoscopic, and genetic features, the article offers a roadmap for clinicians navigating the diagnostic and management challenges posed by these tumors. These tumors primarily affect the face and neck region and are often mistaken for other cutaneous malignancies. Sebaceous tumors can manifest as various lesions, including papules, nodules, or exophytic tumors, and may present with ulceration or other features mimicking common skin cancers. Dermoscopic examination can aid in the diagnosis, which is confirmed through histopathology. While sebaceous carcinomas are rare, they represent a significant proportion of periorbital malignancies. Older age and Asian ethnicity are identified as risk factors, with sebaceous carcinoma being less common in Africans. The exact cause of sebaceous tumors is not fully understood; however, factors such as chronic sun exposure and immunosuppression play a role. Genetic mutations, including those associated with Muir–Torre Syndrome, contribute to the development of these tumors. Sebaceous carcinoma is a malignant tumor that primarily affects the periocular region and can metastasize, particularly in cases of periocular involvement. Diagnosis can be challenging and requires histopathological examination, with immunohistochemistry aiding in distinguishing sebaceous carcinoma from other skin malignancies. Treatment involves surgical excision with margin control, often utilizing techniques such as Mohs micrographic surgery. Adjuvant radiotherapy may be considered in certain cases, particularly with positive margins or perineural invasion. Sebaceoma and sebaceous adenoma are benign tumors that share similar clinical features but differ histopathologically. Dermoscopic examination can aid in the diagnosis, with both lesions typically presenting as nodules with distinctive vascular patterns. Sebaceous hyperplasia is a common proliferative abnormality, characterized by firm, skin-colored papules, primarily affecting the face. Diagnosis is established through histopathology, with total excision recommended for definitive diagnosis and exclusion of other sebaceous neoplasms. Overall, early diagnosis and appropriate management of sebaceous neoplasms are crucial for optimal patient outcomes, particularly in cases associated with Muir–Torre Syndrome.

Lastly, Tsiogka et al. (contribution 5) unravel the complexities surrounding eccrine porocarcinoma (EPC), a rare malignant adnexal tumor with diverse clinical presentations and uncertain prognosis. Through an exhaustive review of epidemiology, pathogenesis, and diagnostic approaches, the article underscores the need for a multidisciplinary approach to managing this rare cutaneous malignancy, which comprises about 0.005–0.01% of all skin cancers. It can arise de novo or from benign eccrine poromas and is classified under the umbrella of eccrine sweat gland tumors. EPC predominantly affects the elderly population, with a mean age of presentation ranging from 63.6 to 66 years. The incidence rate varies between studies but generally falls within the range of 0.02–0.2 cases per 100,000 person-years. The exact cause of EPC is not fully understood; however, it is believed to involve specific oncogenic drivers and signaling pathways, including mutations associated with UV exposure. Risk factors include chronic UV exposure, immunosuppression, and pre-existing skin damage such as from radiotherapy or trauma. Diagnosis of EPC can be challenging due to its variable clinical and histopathological features. It requires a combination of clinical, dermoscopic, histopathological, and immunohistochemical findings. The differential diagnosis includes various benign and malignant skin lesions. EPC typically presents as erythematous or violaceous nodules, often growing over weeks to months. It may be asymptomatic or accompanied by symptoms such as itching, ulceration, or bleeding. Common sites of involvement include the head and neck, lower extremities, and trunk.

In conclusion, this special issue encapsulates the evolving landscape of skin and cutaneous adnexal tumors, offering a wealth of knowledge to guide clinical practice and future research endeavors. We extend our gratitude to all the authors, reviewers, and editorial staff involved in bringing this issue to fruition.
